# Supplementary material for: PLD2 regulates microtubule stability and spindle migration in mouse oocytes during meiotic division
Source: PeerJ. 2017 May 16;5:e3295. doi: 10.7717/peerj.3295 (PMC5436581; doi:10.7717/peerj.3295)

Spindle length of oocytes (L value) after FIPI treatment

Replicates	DMSO	12.5 μM FIPI	25 μM FIPI	37.5 μM FIPI
**1**	0.2031888	0.2166102	0.2343498	0.2034701
**2**	0.2242580	0.2329463	0.2481365	0.2579766
**3**	0.2644219	0.2453822	0.2445720	0.2620143

Declaration: Spindles in MI oocytes were measured and compared.

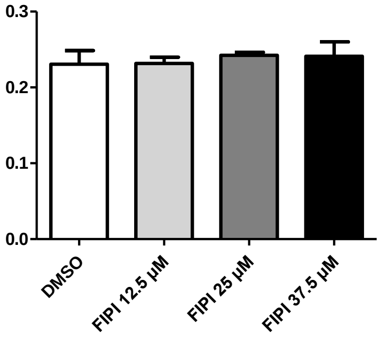

Supplement: Supplemental Information 1 — The spindle size was not affected when oocytes were cultured in different concentrations of FIPI. [file peerj-05-3295-s001.pdf]
