# Supplementary material for: PLD2 regulates microtubule stability and spindle migration in mouse oocytes during meiotic division
Source: PeerJ. 2017 May 16;5:e3295. doi: 10.7717/peerj.3295 (PMC5436581; doi:10.7717/peerj.3295)
Supplement: Data S1 — GV oocytes were cultured for 10 h, 12 h and 14 h, respectively, in maturation medium with NFOT, and accordingly, additional 7 h, 5 h and 3 h in fresh medium without NFOT. Oocytes cultured only with DMSO for 17 h were used as control. After a total 17 h culture , the oocytes matured to MII stage, manifested with extruded 1st PB, were counted. With the decrease in NFOT incubation time, the proportion of MII oocytes was increased. [file peerj-05-3295-s002.pdf]

Supplementary data

S1 NFOT inhibitory effect on oocytes meiotic progression to MII stage was reversible

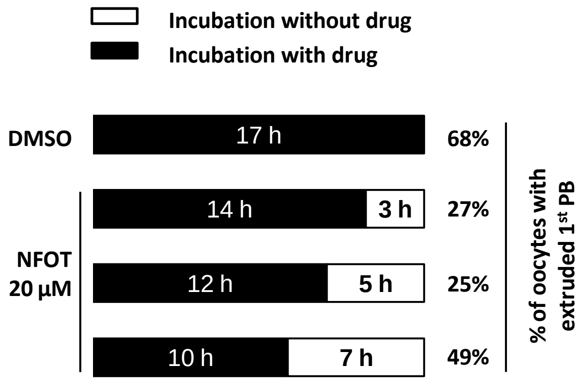

S1 legend: GV oocytes were cultured for 10 h, 12 h and 14 h, respectively, in maturation medium with NFOT, and accordingly, additional 7 h, 5 h and 3 h in fresh medium without NFOT. Oocytes cultured only with DMSO for 17 h were used as control. After a total 17 h culture , the oocytes matured to MII stage, manifested with extruded 1st PB, were counted. With the decrease in NFOT incubation time, the proportion of MII oocytes was increased.
